# Mixed Models as a Tool for Comparing Groups of Time Series in Plant Sciences

**DOI:** 10.3390/plants10020362

**Published:** 2021-02-13

**Authors:** Ioannis Spyroglou, Jan Skalák, Veronika Balakhonova, Zuzana Benedikty, Alexandros G. Rigas, Jan Hejátko

**Affiliations:** 1 Plant Sciences Core Facility, CEITEC—Central European Institute of Technology, Masaryk University, Kamenice 5, 62500 Brno, Czech Republic; 2 Functional Genomics & Proteomics of Plants, CEITEC—Central European Institute of Technology and National Centre for Biotechnology Research, Faculty of Science, Kamenice 5, 62500 Brno, Czech Republic; jan.skalak@ceitec.muni.cz (J.S.); veronika.balakhonova@ceitec.muni.cz (V.B.); hejatko@sci.muni.cz (J.H.); 3 Photon Systems Instruments, (PSI, spol. sr.o.), 66424 Drásov, Czech Republic; benedikty@psi.cz; 4 Department of Electrical and Computer Engineering, Democritus University of Thrace, 67100 Xanthi, Greece; rigas@ee.duth.gr

**Keywords:** *Arabidopsis*, linear mixed models, time series analysis, ARIMA

## Abstract

Plants adapt to continual changes in environmental conditions throughout their life spans. High-throughput phenotyping methods have been developed to noninvasively monitor the physiological responses to abiotic/biotic stresses on a scale spanning a long time, covering most of the vegetative and reproductive stages. However, some of the physiological events comprise almost immediate and very fast responses towards the changing environment which might be overlooked in long-term observations. Additionally, there are certain technical difficulties and restrictions in analyzing phenotyping data, especially when dealing with repeated measurements. In this study, a method for comparing means at different time points using generalized linear mixed models combined with classical time series models is presented. As an example, we use multiple chlorophyll time series measurements from different genotypes. The use of additional time series models as random effects is essential as the residuals of the initial mixed model may contain autocorrelations that bias the result. The nature of mixed models offers a viable solution as these can incorporate time series models for residuals as random effects. The results from analyzing chlorophyll content time series show that the autocorrelation is successfully eliminated from the residuals and incorporated into the final model. This allows the use of statistical inference.

## 1. Introduction

A series of data is considered as a time series when it consists of observations taken or sampled sequentially in time. The most important feature of time series is that the data are not identically and independently distributed (IID) [1]. This feature makes methods such as simple regression models or Analysis of Variance (ANOVA) inappropriate for the analysis of these data. Time series analysis is an important tool for every science. The study of phenomena across time may lead to new important findings for every scientific field. Nevertheless, there are certain difficulties and restrictions in analyzing these kinds of data, especially when there are many repeated measurements. Many repeated measurements used in time series problems usually indicates that the data in the time points for each individual plant will be correlated and not independent (autocorrelation). For example, the value in T_n+1_ directly depends on the value in T_n_. So, methods such as classical ANOVA or t-tests at each time point cannot be used as they assume independence between the measurements which does not hold for time series. To our knowledge, so far, a standard methodology for comparing mean values of data in different groups of time series does not exist. As a result, a combination of methods including time series analysis, which can deal with the special nature of longitudinal time series data, must be applied. Consequently, if we find a valid way to identify and describe the mechanism of creating the data (by fitting an appropriate statistical model), we can proceed to statistical inference and multiple pairwise comparisons of time series data which may lead to biological conclusions. Therefore, we must create a model (a mathematical relationship between the response variable we want to examine and explanatory variables of interest) that takes this kind of correlation into account. Additionally, since we want to compare several time series, we need to find a modeling method that will allow us to incorporate them into one single model. Linear mixed models (LMMs) are the perfect mathematical tools for this situation as they can combine population average (fixed for all individual measurements) effects and subject-specific (called random) effects for each individual plant separately, so that the variability between genotypes and time points is retained. As a result, a combination of linear mixed models and time series analysis is used to address all the above-mentioned issues.

Photosynthetic acclimation to environmental conditions such a fluctuating irradiance is an example of a biological process comprising short-term (minutes- or even seconds-long events including changes in electron transport and enzyme activities, CO_2_ diffusion, nonphotochemical quenching, light harvesting capacity, etc. [2,3,4]) and long-term (events lasting for days such as photosynthetic acclimation) responses [5]. Moreover, these biological processes are species-specific, and depend on environmental factors such as daily and seasonal irradiance fluctuations and circadian rhythms. For more details describing the impact of environmental factors on photosynthesis, we recommend following these comprehensive studies [6,7,8,9,10,11,12]. Plant phenotyping is based on noninvasive high-throughput monitoring of parameters including photosynthetic activity, growth dynamics in response to the environment, etc. [13,14]. As a high-throughput method, phenotyping produces a quantity of longitudinal or time-to-event data which is challenging to process by an adequate statistical approach [15,16,17]. In our study, we show that not considering the dependency that exists among individual time points in time series might lead to wrong assumptions, which might further cause a false hypothesis to be made. On the other hand, we show that using mixed models combined with time series analysis methods as a tool for comparing groups of time series in plant sciences overcomes such an issue.

After germination under the surface of the soil (i.e., in the darkness), the plants start elongating with bended cotyledons forming what are called apical hooks, protecting the plant apex while reaching the soil surface, where the light induces the process of de-etiolation. De-etiolation is by far the most dramatic period of plant life cycle, characterized by complete rearrangement of plant metabolism (i.e., from heterotrophic de-etiolated seedling to autotrophic plant). The process of de-etiolation is (among other things) initiated by very fast (immediate) activation of light-responsive enzymes, involved in the chlorophyll biosynthesis from its dark-phase accumulated precursor protochlorophyllide [18]. This is a very delicate operation for the plant, as chlorophyll must be synthesized very quickly, but the chlorophyll itself or its biosynthesis intermediates can (instead of transferring their excitation energy to excited porphyrin pigments) mediate formation of singlet oxygen, leading to strong photo-oxidative damage of chloroplasts [19]. Thus, sensitive phenotyping systems as well as a correct and precise data processing that allows monitoring of the very fast (taking place in order of minutes or even seconds) and subtle changes in chlorophyll biosynthetic dynamics are required for studies on the processes associated with the early stages of a plant’s life in light (photomorphogenesis). 

The goal of this paper is to find a statistical tool allowing unbiased comparison in the chlorophyll fluorescence dynamics of different genotypes (wild type vs. mutant). This fast biological process serves as an example of time series data, where the dynamic change can take place in the first minutes of observation. More specifically, we seek to find when in time the difference between the chlorophyll fluorescence mean values of the genotypes becomes statistically significant. In this way, we will be able to determine significant early and/or late differences in chlorophyll dynamics which might otherwise be incorrectly identified by a wrong statistical approach. For this reason, we present a combination of methods that can lead to accurate chlorophyll fluorescence results in the case of multiple comparisons of chlorophyll fluorescence in different groups of time series. In particular, linear mixed models (LMMs) [20] are applied in combination with methods of time series analysis which can help us to overcome the difficulties arising from the longitudinal nature of the data. Using this method, the dynamic changes in chlorophyll fluorescence of several de-etiolating genotypes are clearly identified.

## 2. Materials and Methods

### 2.1. Plant Material and Chlorophyll Quantification

The data used in this study consist of 27 time series. We performed fluorescence-based chlorophyll measurements in 3 genotypes of *Arabidopsis thaliana*, and for each genotype we conducted 3 independent experiments, each containing 3 biological replicas. This makes 9 different time series for each genotype. The genotypes are one wild type (WT) Landsberg erecta-1 (Ler-1) and two mutants in the same genetic background defective in chlorophyll biosynthesis (light-dependent protochlorophyllide to chlorophyllide conversion), which were cultivated in vitro for 4 days on half-strength Murashige and Skoog (½MS) medium in the dark (16 h cycle and 8h cycle at 21 and 19 °C, respectively). The same Ler-1 background lines were selected since different genetic backgrounds might be associated with modulated growth and stress responses [21].

The starting point refers to the exposure of the seedlings to the first short pulse of actinic light (50 ms, 238 µmol m^−2^ s^−1^; 21 °C) used to induce fluorescence of the biosynthesized chlorophyll for nondestructive real-time in vivo measurements of chlorophyll content (Balakhonova, Dobisova et al., manuscript in preparation). Briefly, chlorophyll content was quantified using imaging fluorometer FluorCam (Photon Systems Instruments, Drásov, Czech Republic). The fluorescence signal was acquired by a sensitive CCD camera (1.4 M pixels) with an emission band of 690–770 nm. Camera resolution allowed analysis of pixels corresponding to cotyledons of individual seedlings. The fluorescence emission was excited by 50 ms long pulses of blue actinic light generated by a LED light source with a peak wavelength of 470 nm. The pulse was sufficient to induce chlorophyll biosynthesis (de-etiolation), thus no additional source of light was used.

The time series consists of 121 measurements that were taken over a 4 h and the sampling period (defined by the pulse of the actinic light) was every 2 min. 

Data were scaled to the first time point measured (T0), so all variables started at the same point, allowing comparison of time series data which inputs values differing in scale. Every value of the time series was divided by the first corresponding time point. As a result, the first time point was always 1.

**Figure 1:**
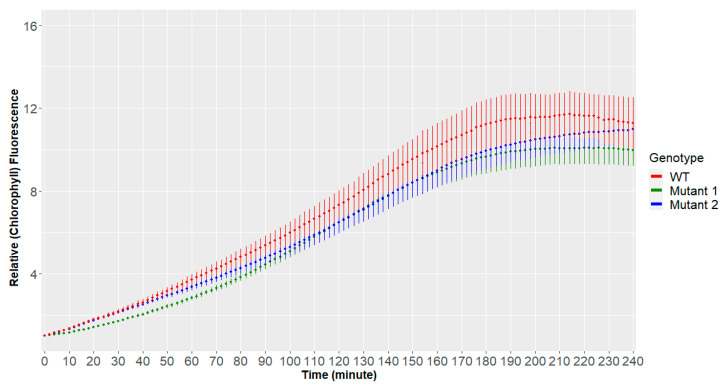
Means (dots) and confidence intervals (error bars) for groups of time series (WT: wild type).

The scaled data are given in Figure 1 where the dots represent the mean of all the time series of the same genotype and the error bar represents a 95% confidence interval.

### 2.2. Linear Mixed Models and Time Series analysis

The idea behind linear mixed models (LMMs) is that different coefficients are estimated for different subjects [20]. The estimates are characterized as subject-specific because they are conditional on the random subject effect. This property makes LMMs appropriate for modeling longitudinal and clustered data [17]. The implementation of a LMM analysis begins with the construction of a normal distribution around the intercepts and then the variance of this normal distribution is estimated. That variance is added to the regression model in order to create different coefficient for every subject. As a result, an extension of the simple linear model of the form:

(1) $$y=X_{i}^{T}b,i=1,\ldots ,N$$

is the following:

(2) $$y_{ij}=b_{0}+X_{ij}^{T}b+u_{0j}+\varepsilon_{ij}$$

where $b_{0}$ is the fixed-average intercept, $u_{0j}$ is the random (subject-specific) intercept for subject j, b is the vector of regression parameters, $X_{ij}$ is the matrix of explanatory variables and $\varepsilon_{ij}$ is the error for subject j at time i.

As can be seen from Equation (2), the intercepts differ between the subjects, but the other regression coefficients are the same for all subjects. Additionally, it is common in longitudinal studies for someone to also include random effects for the remaining coefficients, so that all the coefficients differ between subjects. The resulting model in this case is given by Equation (3):

(3) $$y_{ij}=b_{0}+X_{ij}^{T}b+u_{0j}+Z_{ij}^{T}u_{j}+\varepsilon_{ij}$$

where $u_{j}$ represents the vector of random (subject-specific) coefficients for the variables and $Z_{ij\;}$ is the matrix of explanatory variables associated with $u_{j}$. 

The coefficients can be estimated by using the Maximum Likelihood (ML) and the Restricted Maximum Likelihood (REML) methods. The estimates for the random effects variances obtained by the REML method are less biased, while the ML method allows for comparisons between models with the use of information criteria such as the Akaike Information Criterion (AIC) and Bayesian Information Criterion (BIC), which have no meaning for the REML estimates. For more detail, refer to [22,23]. In this paper the ML method is used to choose the best model describing the data.

When modeling repeated measurements of different subjects (seedlings) using the time t as a variable, we obtain an estimate of the trend of the time series. It is possible that within the residuals $\varepsilon_{ij}$ (error between actual values and model fitted values), autocorrelations remain. More information about the estimate of the autocorrelation function is given in Appendix A [1].

In this case, $\varepsilon_{ij}$ need to be further modeled so they do not contain any kind of autocorrelations and the whole model may be considered valid.

Thus, the final form of the general model used in this work to compare means between different time points can be given by Equations (4) and (5):

(4) $$y_{ij}=b_{0}+X_{ij}^{T}b+u_{0j}+Z_{ij}^{T}u_{j}+\varepsilon_{ij},$$

The ${\hat{\varepsilon }}_{ij}$ are described as:

(5) $$ARIMA\left(p_{j},d_{j},q_{j}\right)\times {\left(P_{j},D_{j},Q_{j}\right)}_{S}$$

The autoregressive integrated moving average (ARIMA) model was used to fit the initial residuals $\varepsilon_{ij}$, while $r_{ij}$ are the final residuals of the whole model. 

In Equation (5), ($p_{j},d_{j},q_{j})$ is the nonseasonal part of the model for each subject and $\left(P_{j},D_{j},Q_{j}\right)$ is the seasonal part of the model if seasonality is present. If seasonality is absent, we ignore the second part. 

Mixed models are ideal for situations with observations that are not identically and independently distributed (IID) and when we have repeated measurements for different individuals (seedlings). Additionally, there is a degree of randomness in the data acquisition that is not fully controllable. The mixed model we propose contains, as random effects, the effects of each seedling separately, so that the model includes the randomness that might exist between experiments and genotypes. As fixed and random parts of the model (initially, before the addition of ARIMA models as random effects), we used the variables Time (1, 2, 3,…, 121) and $Time^{2}\;\left(1,\;4,\;9,\ldots ,121^{2}\right)$. The variable Time represents the time points when the measurements were taken (1st time point is 0 min, 2nd time point is 2 min, 3rd time point is 4 min, etc.). A categorical variable “Genotype” (3 categories: wild type (WT), Mutant 1 (MUT1), and Mutant 2 (MUT2)) was also used, but only in the fixed part of the equation as the random effects already contained the genotypic differences and the model would not converge if we included “Genotype” in the random effects as well. Here, “Genotype” is a dummy variable with the 3 referred categories. The first (reference) category is the WT and its information is contained in the intercept term $b_{0},$ which plays a significant role. 

Thus, MUT1 and MUT2 are binary variables—e.g., MUT1 = 1 when the genotype is MUT1 and 0 otherwise. When MUT1 and MUT2 are both equal to zero, then the genotype is WT. So, the values of coefficients $b_{3}$ and $b_{4}$ show the general differences of MUT1 and MUT2 with the WT. More information about dummy variables can be found in [24]. Finally, the covariance structure that is used with this dataset is the heterogeneous unstructured structure, which is the most commonly used as it does not limit the values of the covariance matrix at all [25].

So, the model of Equation (4) takes the following form (Equation (6)):

(6) $$y_{ij}=b_{0}+b_{1}Time+b_{2}Time^{2}+b_{3}{\mathrm{Genotype}}_{\mathrm{MUT}1}+b_{4}{\mathrm{Genotype}}_{\mathrm{MUT}2}\;+u_{0j}+u_{1j}Time+u_{2j}Time^{2}+{\hat{\varepsilon }}_{ij}+r_{ij}.$$

Instead of using the original values of the time series, we can take their logarithms. It was found in several studies that the log transformation of the underlying series improves forecasting and stabilizes the variance [26].

The modeling procedure and data analysis were performed in R studio (R GUI 4.0.3) with the use of “glmmTMB” and “forecast” packages [27,28,29,30,31].

## 3. Results

The difficulty here follows from the fact that for different subjects the form of the autocorrelation might be different and thus we will need a different model for each subject’s residuals [24,25,26].

In Figure 1, we can observe the means (dots) and the confidence intervals (error bars) for every group of chlorophyll fluorescence time series after transferring the seedlings from the dark to actinic light condition. The goal is to discover if and when in time the difference between the means of the genotypes becomes statistically significant.

To be able to do this, we need to create a common forecasting model that describes all the time series that we want to analyze equally well. The LMM of Equation (6) is found to be the best model to describe these data according to AIC. 

The case of a third order term, $Time^{3},$ was examined as well, but the coefficient was found to be nonsignificant and the AIC was higher. The model of Equation (6) successfully describes the trend of all the time series in one single mixed model. In addition, looking at the coefficients $b_{3}$ and $b_{4}$ of ${\mathrm{Genotype}}_{\mathrm{MUT}1}$ and ${\mathrm{Genotype}}_{\mathrm{MUT}2}$, we have an indication of whether there are differences between WT and MUT1-MUT2 by the significance of the coefficients of dummy variables (MUT1 and MUT2). 

**Figure 2:**
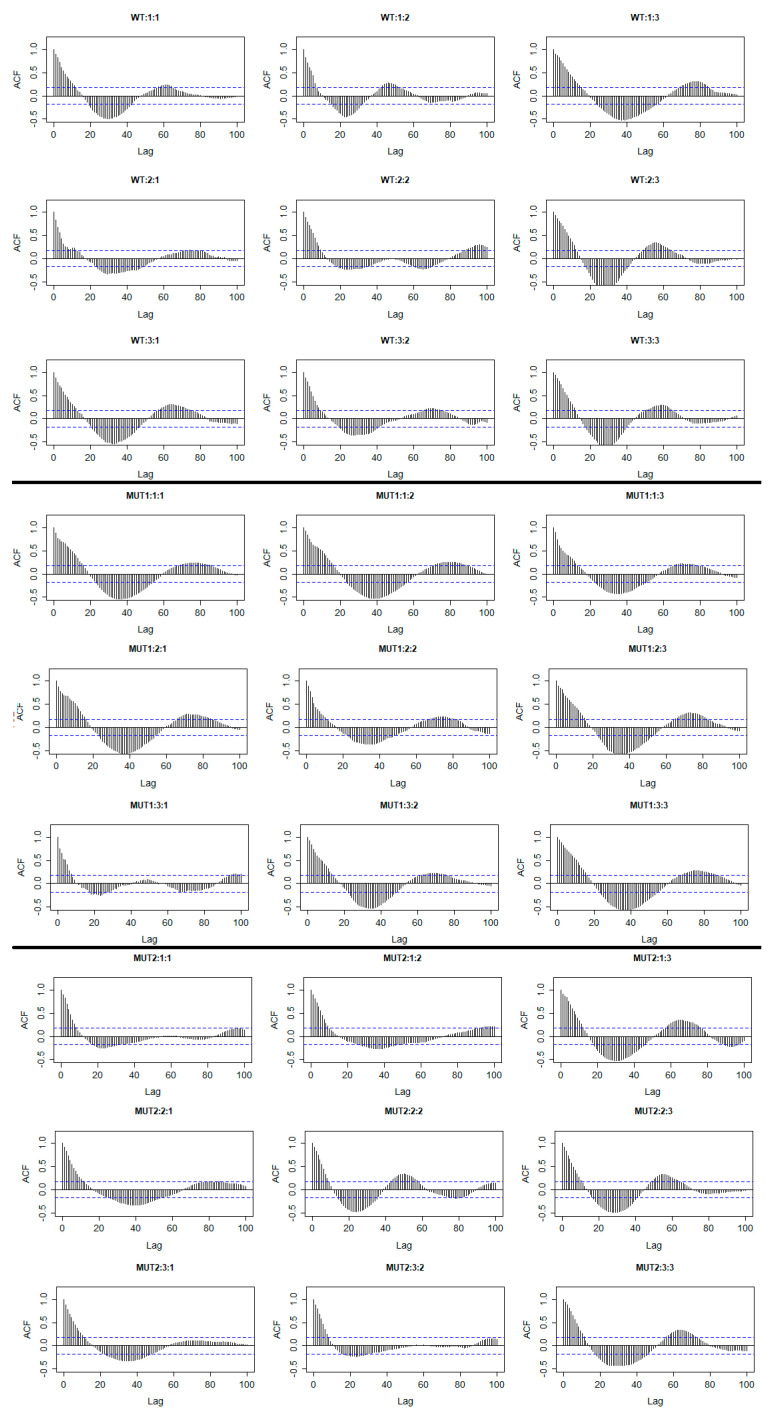
Estimated autocorrelations of the residuals of the initial model given by Equation (3). The title of each plot refers to Genotype: Replicate: Experiment (WT: Wild Type, MUT1: Mutant 1, MUT2: Mutant 2). The dashed blue lines represent the 95% confidence interval. More information about the autocorrelation function is given in Appendix A. It can be observed in the residuals of every time series that there are patterns of significant autocorrelations (several values are outside the 95% confidence interval). This means that further modeling is required to remove these autocorrelations from the residuals.

**Figure 3:**
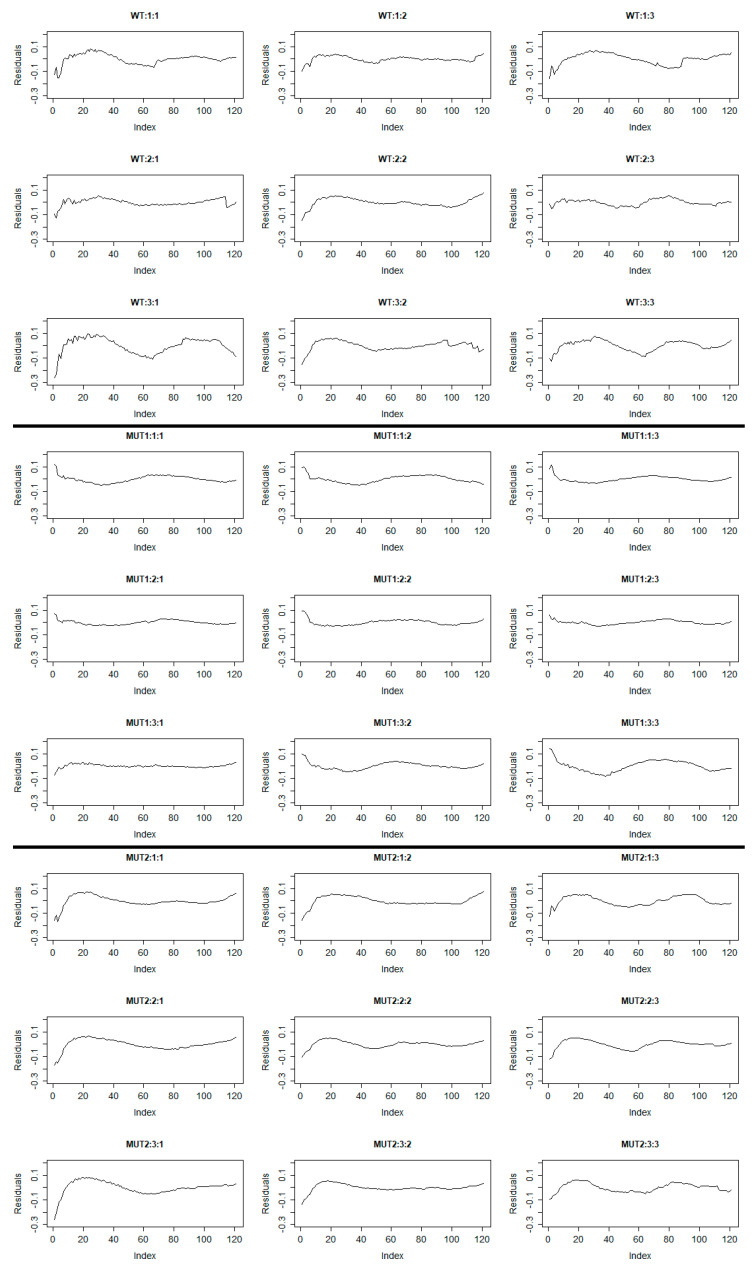
Plot of estimated residuals for each time series of the initial mixed model ($\varepsilon_{ij}$ in Equation (3)). Index refers to time points (2 min, 4 min, etc.). The title of each plot refers to Genotype: Replicate: Experiment (WT: Wild Type, MUT1: Mutant 1, MUT2: Mutant 2). It is obvious that long-term increases or/and decreases (trend) exist in the residuals of the model given by Equation (3).

However, if we plot the autocorrelation function for the residuals of each time series, we will see that strong autocorrelations exist. This means that if we directly apply statistical inference in this model to identify differences (multiple comparisons, etc.), the result will be incorrect. This happens because the model which is used for forecasting is not the correct one, as these autocorrelations should be incorporated in the model fitted values (i.e., they are taken into consideration when we make comparisons) and not in the residuals, as the data here are not independent, as mentioned before, so the inclusion of autocorrelations in the model is essential. Otherwise, we can obtain biased (misleading) results (either false positive or false negative). Estimates of the autocorrelations of residuals for each seedling are given in Figure 2. Autocorrelation plots such as those in Figure 2 show the dependence that exists within observations in every time series. The autocorrelation with lag zero always equals 1, because it reflects the autocorrelation between each observation in the time series and itself. The autocorrelation with lag 1 shows the relation that exists between the observations of the time series whose distance is one step; the one with lag 2 shows the relation that exists between the observations whose distance is two steps, etc. Each value of the autocorrelation function that is above or below the dashed lines (95% confidence interval) is considered to be statistically significant. If we have values significantly different from zero, this shows that significant autocorrelations exist in the time series. Briefly, significant autocorrelations contain information that should be included in the model and not in the residuals of the model. If the residuals of the model do not contain significant autocorrelations, the procedure described in this work is not necessary. In Figure 2, one can observe that the chlorophyll measurements are highly correlated with each other as there are many values of the autocorrelation functions above and below the 95% confidence intervals. To be able to remove these autocorrelations from the residuals and make the model appropriate for statistical inference, time series analysis can be applied. One way to do this is to separately examine every autocorrelation function and find a proper time series model to remove it. The most popular time series models for this case are the autoregressive (AR) models, the moving average (MA) models, and the more complex autoregressive moving average (ARMA) models or autoregressive integrated moving average models (ARIMA) in case trends are still present in the residuals. It is possible to have more complex time series where periodical phenomena are present. In this case, seasonal ARIMA (SARIMA) models can be useful [32]. A more automatic procedure that makes things easier and much faster is the “auto.arima” function from the “forecast” package in R. The auto.arima function uses a variation of the Hyndman–Khandakar algorithm, which combines unit root tests and finds the best ARIMA model based on several information criteria such as AIC, AICc (corrected AIC for small samples), and BIC [28]. Plotting the residuals will allow us to see if trends remain. Figure 3 presents the residuals of the initial model of Equation (3). A trend is present in time series when a long-term increase or decrease exists. Identifying these patterns is the first step for choosing a proper forecasting method. Figure 3 clearly shows that trends are still present in the residuals. 

**Figure 4:**
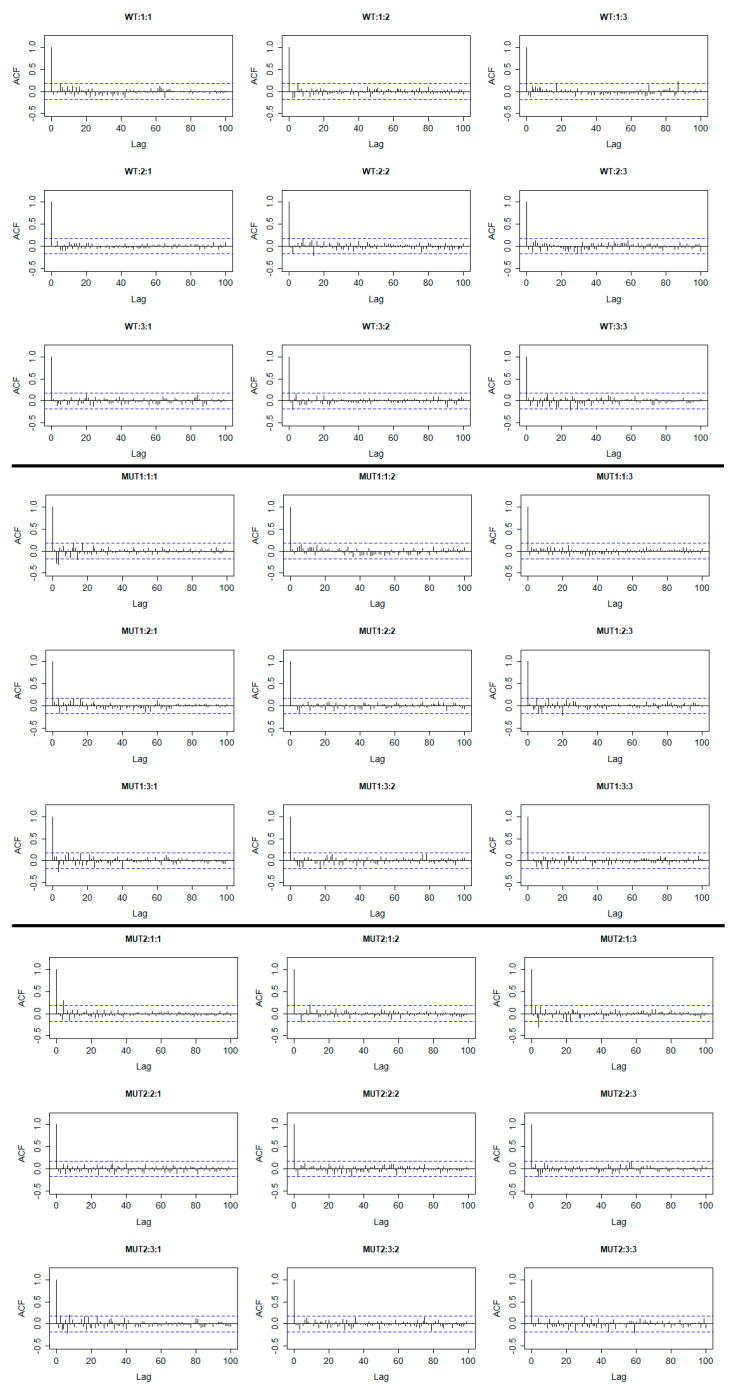
Estimated autocorrelations of the final residuals $r_{ij}.$ The title of each plot refers to Genotype: Replicate: Experiment. The dashed blue lines represent the 95% confidence interval. (WT: Wild Type, MUT1: Mutant 1, MUT2: Mutant 2). We can see that except for the zero lag which is always 1, there are generally no statistically significant values (outside the 95% confidence intervals). This means that the model is now valid for statistical inference.

To deal with this, we need to set at least d=1 in the ARIMA models for all the residuals, so the trends were eliminated from the time series after differentiation. In Equation (5), $d_{j}$ reflects the number of differences (nonseasonal) needed for the trends to be removed. Differentiation in time series might help to stabilize the mean value of the time series and it is the most popular way of removing trends [32]. A list of all the fitted ARIMA models for the residuals is provided as a supplementary material. Finally, if we add these models to the previously fitted LMM, we obtain the form of the model given in Equation (4). It is obvious from Figure 4 that the estimated residuals $r_{ij}$ of the final model given by Equation (4) and consequently by Equation (6) do not contain significant autocorrelations as there are no significant values in general. This implies that the model is now valid for statistical inference. Additionally, the mean values of the residuals for each genotype are very close to zero which is another important result (see Supplementary Materials: Figure S1).

Furthermore, other diagnostics for the model are provided as Supplementary Materials (Figures S2 and S3).

Hereafter, we can proceed with the comparison of the means of each time point. Since in R there is no automatic procedure for such a model yet, we can use a t-test considering the Bonferroni correction for all time points. At each time point are three comparisons, so that the corrected threshold for the *p*-value is 0.05/3 = 0.01667. The t-test was applied on the model fitted values and the groups that are compared are the values (scaled) of chlorophyll at separate time points between genotypes. For example, for the first time point, we compared the mean value of WT chlorophyll measurement with the MUT1 mean value. It is very critical to correct the *p*-value, otherwise there will be an increase in Type I errors [33].

The fixed part of the model is given in Table 1. The “Estimate” column corresponds to $b_{0},\;b_{1},\;b_{2},\;b_{3}$ and $b_{4}$ of Equation (6). Additionally, the standard deviation that exists within the random/subject-specific effects ($u_{0j},\;u_{1j}$ and $u_{2j})$ is given. The random effects $u_{0j},\;u_{1j}$ and $u_{2j}$, as well as those concerning the residuals, are provided as supplementary materials (the standard deviation in Table 1 resulted from finding the standard deviation for each column of the supplementary file random_effects.xlsx which corresponds to the values of $u_{0j},\;u_{1j}$ and $u_{2j}$ of Equation (6)). With regard to the fixed coefficients of MUT1and MUT2, we expected, in the final analysis, to see significant differences between WT-MUT1 (*p*-value of coefficient of MUT1 is <0.001 which indicates significant difference from the reference genotype WT) and MUT1-MUT2 (MUT2 is not significantly different from WT (*p*-value = 0.657)). However, it would be wrong to make inferences just from these p-values as the random effects of the ARIMA models are not considered and they might significantly affect this result in the pairwise comparisons, as referred to before, because of the autocorrelation.

Thus, based on this modified model combined with the ARIMA models for the residuals (see supplementary file random_effects.xlsx and ARIMA_models.pdf), we can proceed to apply statistical inference.

**Figure 5:**
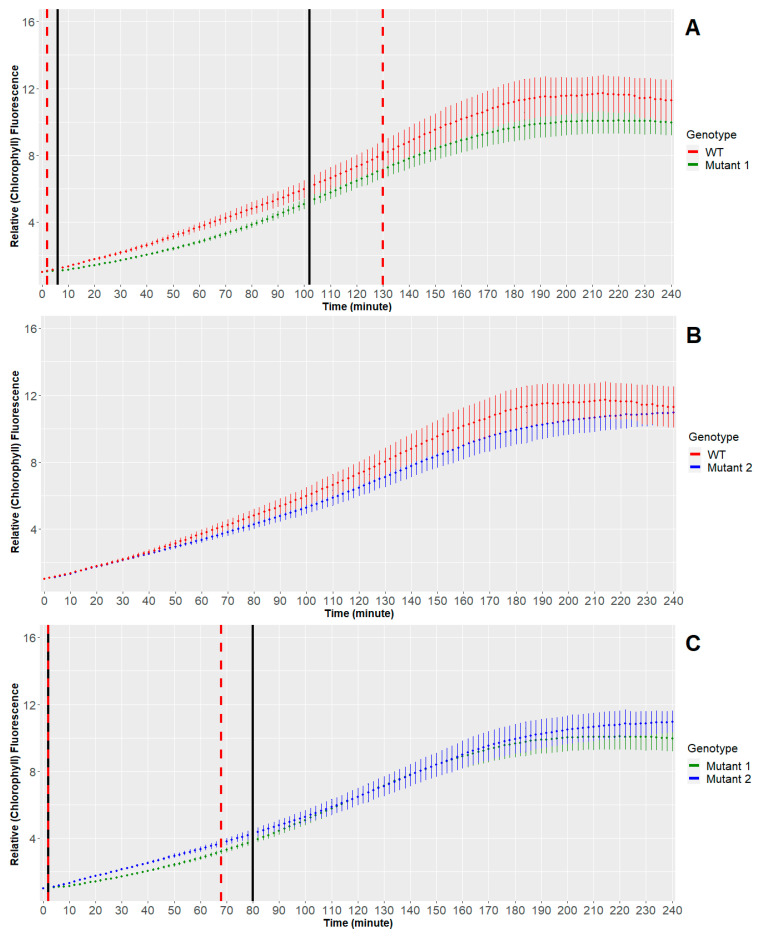
Pairwise comparisons of genotypes for each time point. The intervals inside the solid black vertical lines are considered as significantly different. The intervals inside the dashed red lines are the wrong results we would have if we did not take the autocorrelation into consideration. (**A**) There are significant differences in the chlorophyll dynamics between WT and Mutant 1 from 6 min to 102 min after the first measurement. Without considering autocorrelations, the (wrong) result would be from 2 min to 130 min (dashed red lines). (**B**) There are no significant differences found between WT and Mutant 2. (**C**) There are significant differences in the chlorophyll dynamics between Mutant 1 and Mutant 2 from 2 min to 80 min after the first measurement. Without considering autocorrelations the (wrong) result would be from 2 min to 68 min (dashed red lines).

The multiple comparison results are summarized in Figure 5. The vertical lines correspond to the intervals in which there are significant differences between the genotypes. Indeed, as indicated in Table 1, we obtained significant differences between WT-MUT1 and MUT1-MUT2.

In the final residuals of the model ($\varepsilon_{ij}$ in Equation (6)), we can see that there are some outliers (see Figure S2: Q-Q plots in Supplementary Materials) which are due to slightly larger prediction errors in the beginning of some of the time series (see Figure S1: Final residuals). If these outliers are not considered, then the residuals approximate the standard normal. These errors exist because the exponential increase is affected by complex regulations in chlorophyll metabolism [18].

## 4. Discussion

Comparing groups of time series can be essential in plant sciences. Longitudinal studies are now very popular in plant biology to describe plant adaptation strategies to various environmental conditions. There are many plants and crop studies containing growth curve modeling during stress and recovery periods. Other methods such as paired t-tests and repeated measures ANOVA (RMANOVA) are useful but not for the type of problem we deal with in this work [34]. A paired t-test (dependent sample t-test) is used to determine if the difference of the means between two groups of observations is zero (null hypothesis) [35]. In a paired sample t-test, each subject or entity is measured twice, resulting in pairs of observations. In an RMANOVA, we can extend this to more time points. These methods can lead to useful conclusions as well. For example, we can check if the genotypes differ if we average over all seedlings and all time points, or if the mean response differs over time when we average over all seedlings and all genotypes. Finally, based on interaction terms, we can assess whether the pattern across time depends upon the specific genotypes. Neither of the above methods can focus on specific time points. Other studies also deal with chlorophyll fluorescence data using time series. In [36], the authors identified differences between wild types and mutants using deep neural networks and discriminant analysis. More specifically, they used a time series deep learning algorithm to extract time series chlorophyll fluorescence features which were then used for classification by applying several methods. Their method appeared to be very efficient as the discrimination–classification accuracy percentages are very high. However, this leads to more general results (finds discrimination between mutants and wild type) and not time-specific results as our method obtained. Furthermore, in [37], the author used residuals (restricted) maximum likelihood (REML) models for comparing time series of chlorophyll fluorescence measurements. There is no reference, however, to autocorrelation. The reason for this is that the measurements were taken 0, 23, 47, 71, 143 and 191 h following detachment. When the sampling intervals are so large, and the number of the measurements so small (six measurements with intervals of 24 hours between them), the observations within time series are not correlated and therefore can be considered independent. In contrast, we made 121 measurements with intervals of 2 min between them. Now, if we did not take into account the autocorrelation that exists in the residuals and directly apply statistical inference in the model of Equation (3) (similarly to [37]), i.e., without further analyzing the residuals and removing them from the autocorrelation, we would have obtained false significant differences (i.e., false positives) or/and significant differences which were missed (false negatives). This is so because the autocorrelation contains information that should be included in the estimations of the coefficients of the model and not in the residuals. In particular, for WT-MUT1 (Figure 5A), we would have wrong significant differences until the point of 130 min after the first measurement, and for MUT1-MUT2, we would have 6 points (12 min) which, with the present method, would be considered significant, while without the extra time series analysis, the significance in the difference is lost (false negatives). The wrong results we would have if we did not consider the autocorrelation are shown in Figure 5A,C with dashed red lines. It should be also noted here that, in our study, we did not measure the chlorophyll fluorescence to quantify the photosynthesis efficiency, used most frequently to evaluate the immediate physiological status of the plant as referred to in the mentioned works [36,37]. Here, the chlorophyll fluorescence corresponds dominantly to the chlorophyll amount in the de-etiolating seedlings.

The proposed method, combining LMMs which are commonly used for longitudinal studies, with time series analysis seems to be a valuable tool for analyzing longitudinal data without producing any bias in the results since every case of possible bias due to repeated measurements (non-IID, strong autocorrelations that remain in the residuals, etc.) has been carefully taken into consideration.

The pairwise comparison of genotypes using mixed models identified significant differences in the early phases of de-etiolation (see Figure 5) after transferring the seedlings from dark to light conditions. More specifically, from the multiple pairwise comparisons, the 2-minute interval measurements provided us with sufficient coverage of time points and the final shape of the chlorophyll biosynthetic curves. Thus, we were able to apply our robust statistical method that projected the significant changes in early dynamics of chlorophyll biosynthesis, which could be otherwise obscured by not fulfilling all the necessary conditions for time series analysis. 

To maintain the equilibrium between the fast chlorophyll biosynthesis and avoidance of photo-oxidative damage, plants evolved an instrumentation with delicate and complex regulations, employing feed-forward and feed-back regulatory mechanisms [18,38,39]. This is, however, a rather common feature of many fast-responding (not only) biological systems. To study these complex regulations, we need to measure not only the end points, but also the dynamics of the process before they are reached. For this, we need techniques that can discriminate changes in the measured variable with not only (high) spatial resolution, but also time resolution. A prerequisite of correct data interpretation is using the proper tools for the generated data processing/evaluation. Based on our results, it seems that linear mixed models combined with time series analysis might be one of the possible solutions. One possible limitation of this study is that the model used can describe data that are expressing an exponential increase. In this study, all the genotypes have an exponential increase in chlorophyll through time. In general, in case of applied stress for example, this can change. The problem is not so important when they do not have exponential growth (other models may be applied in this case, e.g., linear, without the use of the exponential term or even logistic models, Gompertz, monomolecular, etc. [40,41]), but when the genotypes that we have to compare follow completely different growth patterns. The modeling process will then become more difficult as we will have to experiment with random effects to find the most suitable model that describes the data and then apply statistical inference. Of course, the method used in this work can be also applied to other scientific fields and areas in which time and repeated measurements play important roles, such as economic and sales forecasting, medical studies, engineering problems and others [1].

## 5. Conclusions

The use of proper statistical methods for data analysis is essential in life sciences. In this work, we present a combination of linear mixed models and time series analysis permitting the use of statistical inference when comparing chlorophyll contents in groups of time series that belong to different genotypes. Even though repeated measurements and longitudinal data are becoming more popular in life sciences as they can examine various effects through time, to our knowledge there is no standard methodology for comparing mean values of time points in different groups of time series. Especially when the time points examined are many, it is almost certain that autocorrelations will be present within time series and this is not considered for standard models. As a result, a combination of methods must be applied for valid statistical inference.

The results of the analysis combining linear mixed models and time series analysis models show that the proposed method can be a valuable tool to explore fast responses assayed using time series. More specifically, it was found that the significant differences between the wild type and Mutant 1 exist from 6 min to 102 min after the first measurement and between Mutant 1 and Mutant 2 from 2 min to 80 min after the first measurement. No significant difference was found between wild type and Mutant 2. In addition, the results without considering the autocorrelation are provided. These show that if we do not consider this, the results will be different, leading to false positive or/and false negative results.

For future research, it would be interesting to also apply this method under different conditions (stress, heat, etc.) for other phenotypic traits which will be more difficult due to the different dynamics that the genotypes might have under different treatments.

## Figures and Tables

**Table 1:** Linear mixed model for chlorophyll data.

Family = Gaussian (link = Identity)					
Model formulae are given in Equations (4) and (5)					
Fixed Effects:					
Coefficients	Estimate	Standard Error		*z*-Value	*p*-Value
Intercept − $b_{0}$	0.09175	0.0182		5.04	<0.001
$Time-b_{1}$	0.04297	8.471 × 10^−4^		50.72	<0.001
$Time^{2}-b_{2}$	−1.961 × 10^−4^	5.681 × 10^−6^		−34.52	<0.001
$Genotype_{MUT1}-b_{3}$	−0.1914	0.02289		−8.36	<0.001
$Genotype_{MUT2}-b_{4}$	0.1083	0.02443		−0.44	0.657
Random Effects (Conditional Model):					
Groups	Name	Variance	Standard Deviation	Correlation	
Id	Intercept	0.003901	0.0625		
	Time	1.923 × 10^−5^	4.385 × 10^−3^	−0.65	
	$Time^{2}$	8.624 × 10^−10^	2.937 × 10^−5^	0.58	−0.97
Residual		1.318 × 10^−3^	3.630 × 10^−2^		
Number of Groups (id): 27 (3 experiments each containing 3 replicas for each genotype)					
Number of Observations: 3267					

## Data Availability

The data presented in this study are available in supplementary material.

## Supplementary Materials

The following are available online at https://www.mdpi.com/2223-7747/10/2/362/s1, Figure S1: Final residuals of the model, Figure S2: Normal Q-Q plots of the final residuals of the model, ts_means_comparison.R: The R code used for this paper, guide for R code.pdf: Instructions for how to use the R code. Chlorophyll.xlsx: The raw data used in this paper (chlorophyll measurements are denoted as CHL). Random_effects.xlsx: Random effects of the mixed model. ARIMA_models.pdf: ARIMA models for the initial residuals.

## Appendix A

Autocorrelation is defined as the correlation of a signal with a copy of itself and is a function of delay (lag). At lag k≥0 the autocorrelation function is given by Equation (A1):

(A1) $$r_{k}=\frac{\sigma_{k}}{\sigma_{0}},$$

where $\sigma_{0}$ is the estimated variance of the time series and $\sigma_{k}$ is the estimated autocovariance function given by Equation (A2):

(A2) $$\sigma_{k}=\frac{1}{n}\overset{n-k}{\underset{i=1}{\sum }}(y_{i}-\bar{y})\left(y_{i+k}-\;\bar{y}\right)=\frac{1}{n}\overset{n}{\underset{i=k+1}{\sum }}(y_{i}-\bar{y})\left(y_{i-k}-\;\bar{y}\right)$$
