# Supplementary material for: Mixed Models as a Tool for Comparing Groups of Time Series in Plant Sciences
Source: Plants (Basel). 2021 Feb 13;10(2):362. doi: 10.3390/plants10020362 (PMC7918370; doi:10.3390/plants10020362)
Supplement: Supplementary file 1 [file plants-10-00362-s001.zip › Supplementary material/Arima_models.pdf]

```
Series: r1
ARIMA(0,1,0)

sigma^2 estimated as 0.0002412:  log likelihood=329.52
AIC=-657.03   AICc=-657   BIC=-654.25
```

```
Series: r1
ARIMA(1,1,1)

Coefficients:
         ar1      ma1
      0.9483  -0.8304
s.e.  0.0651   0.0814

sigma^2 estimated as 7.934e-05:  log likelihood=397.06
AIC=-788.11   AICc=-787.91   BIC=-779.75
```

```
Series: r1
ARIMA(1,1,0)

Coefficients:
          ar1
      -0.1852
s.e.   0.0920

sigma^2 estimated as 0.000208:  log likelihood=338.88
AIC=-673.76   AICc=-673.65   BIC=-668.18
```

```
Series: r1
ARIMA(1,1,1)

Coefficients:
         ar1      ma1
      0.9891  -0.7589
s.e.  0.0152   0.0560

sigma^2 estimated as 3.811e-05:  log likelihood=440.28
AIC=-874.56   AICc=-874.35   BIC=-866.19
```

```
Series: r1
ARIMA(3,1,2)

Coefficients:
         ar1      ar2     ar3      ma1     ma2
      0.9279  −0.2426  0.2906  −1.1280  0.4984
s.e.  0.2329   0.1702  0.1480   0.2378  0.1843

sigma^2 estimated as 0.0002866:  log likelihood=320.39
AIC=−628.77   AICc=−628.03   BIC=−612.05
```

```
Series: r1
ARIMA(2,1,2)

Coefficients:
          ar1     ar2     ma1      ma2
      -0.0172  0.9484  0.1288  -0.7308
s.e.   0.0533  0.0521  0.0860   0.0810

sigma^2 estimated as 0.0001001:  log likelihood=383.65
AIC=-757.31   AICc=-756.78   BIC=-743.37
```

```
Series: r1
ARIMA(3,1,1)

Coefficients:
          ar1     ar2     ar3     ma1
      -0.5605  0.5392  0.7512  0.7223
s.e.   0.1003  0.1175  0.1068  0.1011

sigma^2 estimated as 5.069e-05:  log likelihood=423.84
AIC=-837.68   AICc=-837.16   BIC=-823.74
```

```
Series: r1
ARIMA(2,1,2)

Coefficients:
         ar1      ar2      ma1     ma2
      1.1879  -0.7419  -1.0338  0.9297
s.e.  0.0917   0.0907   0.0404  0.0913

sigma^2 estimated as 3.057e-05:  log likelihood=454.29
AIC=-898.58   AICc=-898.05   BIC=-884.64
```

```
Series: r1
ARIMA(0,1,0)

sigma^2 estimated as 3.555e-05:  log likelihood=444.39
AIC=-886.79   AICc=-886.76   BIC=-884
```

```
Series: r1
ARIMA(5,1,0)

Coefficients:
         ar1     ar2     ar3      ar4     ar5
      0.2812  0.0662  0.3404  -0.2141  0.2355
s.e.  0.0901  0.0969  0.0863   0.1002  0.1301

sigma^2 estimated as 3.044e-05:  log likelihood=455.77
AIC=-899.55   AICc=-898.8   BIC=-882.82
```

```
Series: r1
ARIMA(1,1,2)

Coefficients:
         ar1      ma1     ma2
      0.9844  -1.4185  0.5558
s.e.  0.0223   0.1095  0.1027

sigma^2 estimated as 4.096e-05:  log likelihood=436.37
AIC=-864.75   AICc=-864.4   BIC=-853.6
```

```
Series: r1
ARIMA(2,1,1)

Coefficients:
         ar1     ar2      ma1
      0.7449  0.1834  -0.6615
s.e.  0.1974  0.1246   0.1959

sigma^2 estimated as 3.139e-05:  log likelihood=453.02
AIC=-898.04   AICc=-897.69   BIC=-886.89
```

```
Series: r1
ARIMA(3,1,1)

Coefficients:
          ar1     ar2     ar3     ma1
      -0.6081  0.3517  0.6966  0.5475
s.e.   0.1964  0.1193  0.1095  0.2101

sigma^2 estimated as 7.309e-05:  log likelihood=402.14
AIC=-794.29   AICc=-793.76   BIC=-780.35
```

```
Series: r1
ARIMA(2,1,1)

Coefficients:
         ar1     ar2      ma1
      0.7800  0.2034  -0.6281
s.e.  0.1405  0.1330   0.1102

sigma^2 estimated as 2.949e-05:  log likelihood=456.09
AIC=-904.18   AICc=-903.83   BIC=-893.03
```

```
Series: r1
ARIMA(3,1,1)

Coefficients:
          ar1     ar2     ar3     ma1
      -0.6046  0.5358  0.7298  0.8251
s.e.   0.0809  0.0744  0.0710  0.0756

sigma^2 estimated as 3.001e-05:  log likelihood=455.25
AIC=-900.51   AICc=-899.98   BIC=-886.57
```

```
Series: r1
ARIMA(1,1,1)

Coefficients:
         ar1      ma1
      0.9831  -0.6308
s.e.  0.0210   0.0677

sigma^2 estimated as 1.313e-05:  log likelihood=504.21
AIC=-1002.42   AICc=-1002.21   BIC=-994.06
```

```
Series: r1
ARIMA(2,1,2) with drift

Coefficients:
         ar1      ar2      ma1     ma2   drift
      1.3688  -0.3762  -1.4623  0.7666  0.0092
s.e.  0.1666   0.1667   0.1173  0.0864  0.0186

sigma^2 estimated as 4.979e-05:  log likelihood=424.64
AIC=-837.28   AICc=-836.53   BIC=-820.55
```

```
Series: r1
ARIMA(3,1,3)

Coefficients:
          ar1     ar2     ar3     ma1     ma2      ma3
      -0.3363  0.3444  0.9510  0.6107  0.0145  -0.4645
s.e.   0.0622  0.0548  0.0468  0.1158  0.1133   0.1062

sigma^2 estimated as 1.853e-05:  log likelihood=484.54
AIC=-955.07   AICc=-954.07   BIC=-935.56
```

```
Series: r1
ARIMA(0,1,0)

sigma^2 estimated as 0.0002109:  log likelihood=337.57
AIC=−673.15   AICc=−673.11   BIC=−670.36
```

```
Series: r1
ARIMA(0,1,0)

sigma^2 estimated as 7.978e-05:  log likelihood=395.9
AIC=-789.8   AICc=-789.77   BIC=-787.01
```

```
Series: r1
ARIMA(1,1,2)

Coefficients:
         ar1      ma1     ma2
      0.8866  -1.0515  0.3887
s.e.  0.0789   0.1026  0.0966

sigma^2 estimated as 9.013e-05:  log likelihood=389.73
AIC=-771.46   AICc=-771.11   BIC=-760.31
```

```
Series: r1
ARIMA(2,1,2)

Coefficients:
          ar1      ar2     ma1     ma2
      -1.3212  -0.4212  1.8896  0.9554
s.e.   0.1075   0.1080  0.0629  0.0605

sigma^2 estimated as 3.777e-05:  log likelihood=441.41
AIC=-872.82   AICc=-872.29   BIC=-858.88
```

```
Series: r1
ARIMA(3,1,1)

Coefficients:
         ar1      ar2     ar3     ma1
      0.3367  -0.0560  0.5087  -0.480
s.e.  0.1855   0.1021  0.0986   0.177

sigma^2 estimated as 2.281e-05:  log likelihood=472.55
AIC=-935.09   AICc=-934.57   BIC=-921.16
```

```
Series: r1
ARIMA(1,1,5)

Coefficients:
         ar1      ma1     ma2     ma3      ma4     ma5
      0.9611  -0.8875  0.1800  0.1729  -0.4333  0.2634
s.e.  0.0474   0.1053  0.1164  0.1279   0.1125  0.1598

sigma^2 estimated as 4.264e-05:  log likelihood=435.77
AIC=-857.54   AICc=-856.54   BIC=-838.03
```

```
Series: r1
ARIMA(3,1,1)

Coefficients:
         ar1      ar2     ar3      ma1
      0.7063  -0.6590  0.7947  -0.4331
s.e.  0.2023   0.1445  0.0928   0.1794

sigma^2 estimated as 7.558e-05:  log likelihood=399.71
AIC=-789.42   AICc=-788.89   BIC=-775.48
```

```
Series: r1
ARIMA(4,1,2)

Coefficients:
         ar1      ar2     ar3     ar4      ma1     ma2
      1.1200  -0.7344  0.1634  0.3605  -1.0060  0.9050
s.e.  0.1034   0.1383  0.1509  0.1203   0.0646  0.0521

sigma^2 estimated as 2.282e-05:  log likelihood=472
AIC=-929.99   AICc=-928.99   BIC=-910.48
```

```
Series: r1
ARIMA(1,1,1)

Coefficients:
         ar1      ma1
      0.9374  -0.7311
s.e.  0.0500   0.0795

sigma^2 estimated as 4.237e-05:  log likelihood=434.6
AIC=-863.2   AICc=-862.99   BIC=-854.84
```
