# Supplementary material for: Mixed Models as a Tool for Comparing Groups of Time Series in Plant Sciences
Source: Plants (Basel). 2021 Feb 13;10(2):362. doi: 10.3390/plants10020362 (PMC7918370; doi:10.3390/plants10020362)
Supplement: Supplementary file 1 [file plants-10-00362-s001.zip › Supplementary material/Guide for R code.pdf]

plants

Mixed models as a tool for comparing dynamic changes in chlorophyll fluorescence between different genotypes

TIPS for the supplementary file tsmeanscomparison.R for scientists not experienced with R

For any queries contact corresponding author: ioannis.spyroglou@ceitec.muni.cz

- Before you run the code, you need to install all the required R packages. This can be done by the following command (you can copy-paste it on the command line - console):

  install.packages(c("openxlsx","glmmTMB","forecast"))

- In line 9 of the code there is the following command:
  - df<-read.xlsx("Chlorophyll.xlsx",colNames = T)

    You can replace the name of the file (Chlorophyll.xlsx) with your own. The names of the columns must be the same as in Chlorophyll.xlsx. The default directory is usually documents but you can use the command getwd() (already included in the code) to find exactly where the working directory is.
- In line 31 there is the following command:

  mdl<-glmmTMB(log(CHL)~1+Genotype+T1+T2+(1+T1+T2|id),data=df, REML = F)

  Here the form of the model log(y)=a0+a1T1+a2*T2 is describing a bellshaped curve.

  If you want to use a linear model (in case the data have different pattern) then this becomes:

  mdl<-glmmTMB(CHL~1+Genotype+T1+(1+T1|id),data=df, REML = F)

  Only exponential increase without the decay second order term:

  mdl<-glmmTMB(log(CHL)~1+Genotype+T1+(1+T1|id),data=df, REML = F)

  etc….

- If you want to compare models to see which one is the best fit use the AIC (and BIC) which is given in summary of the model [line 32 of the code: summary(mdl)]

  The model with the smallest AIC value is the best. For the AIC to have meaning we have to use maximum likelihood estimation (REML=F). Alternatively the command AIC(mdl) can be used.

-

- If the initial residuals do not contain any trend you may delete d=1 in line 58.
- In lines 74, 80, 87: Inside the "for" loops replace "WT" "MUT1" "MUT2" with your genotypes' names on the dataset. If you have more than 3 genotypes include more loops in the same way for all the comparisons.

  For example: If we has also a 3rd mutant it would be like:

```
for (i in 1:tp){

  t<- t.test(df$fit[which(df$Genotype=="WT" &
df$Time==i)],df$fit[which(df$Genotype=="MUT1" & df$Time==i)])
  test[i]<-t$p.value
}
test2<-0
for (i in 1:tp){

  t<- t.test(s[which(df$Genotype=="WT" & df$Time==i)],s[which(df$Genotype=="MUT2" &
df$Time==i)])
  test2[i]<-t$p.value
}
test3<-0
for (i in 1:tp){

  t<- t.test(s[which(df$Genotype=="WT" & df$Time==i)],s[which(df$Genotype=="MUT3" &
df$Time==i)])
  test3[i]<-t$p.value
}



test4<-0
for (i in 1:tp){

  t<- t.test(s[which(df$Genotype=="MUT1" & df$Time==i)],s[which(df$Genotype=="MUT2" &
df$Time==i)])
  test4[i]<-t$p.value
}
test5<-0
for (i in 1:tp){

  t<- t.test(s[which(df$Genotype=="MUT1" & df$Time==i)],s[which(df$Genotype=="MUT3" &
df$Time==i)])
  test5[i]<-t$p.value
}
test6<-0
for (i in 1:tp){

  t<- t.test(s[which(df$Genotype=="MUT2" & df$Time==i)],s[which(df$Genotype=="MUT3" &
df$Time==i)])
  test6[i]<-t$p.value
}
```

And continue likewise.
