# Supplementary figures and images for: Mixed Models as a Tool for Comparing Groups of Time Series in Plant Sciences
Source: Plants (Basel). 2021 Feb 13;10(2):362. doi: 10.3390/plants10020362 (PMC7918370; doi:10.3390/plants10020362)

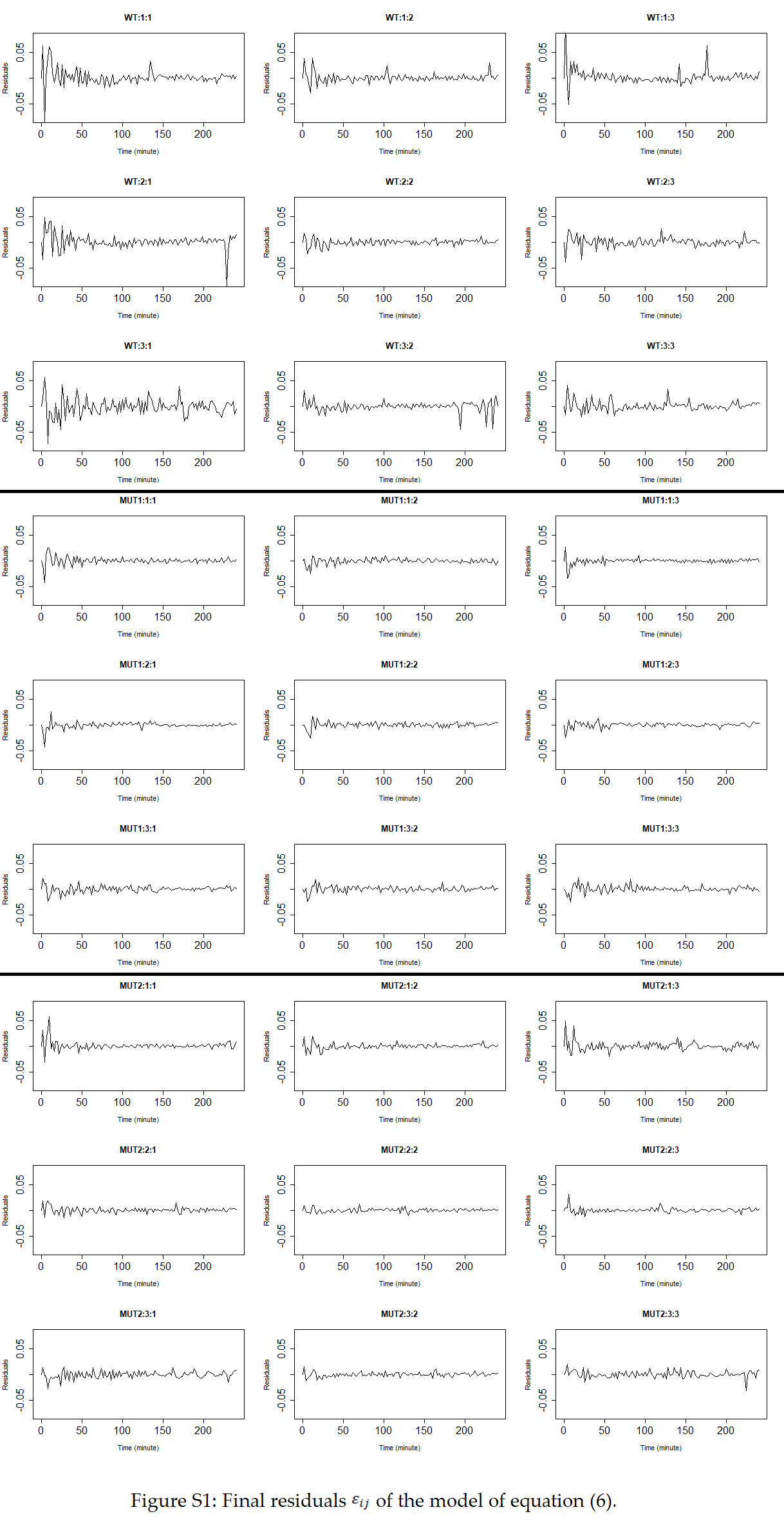

Supplement: Supplementary file 1 [file plants-10-00362-s001.zip › Supplementary material/FigureS1NEW.png]

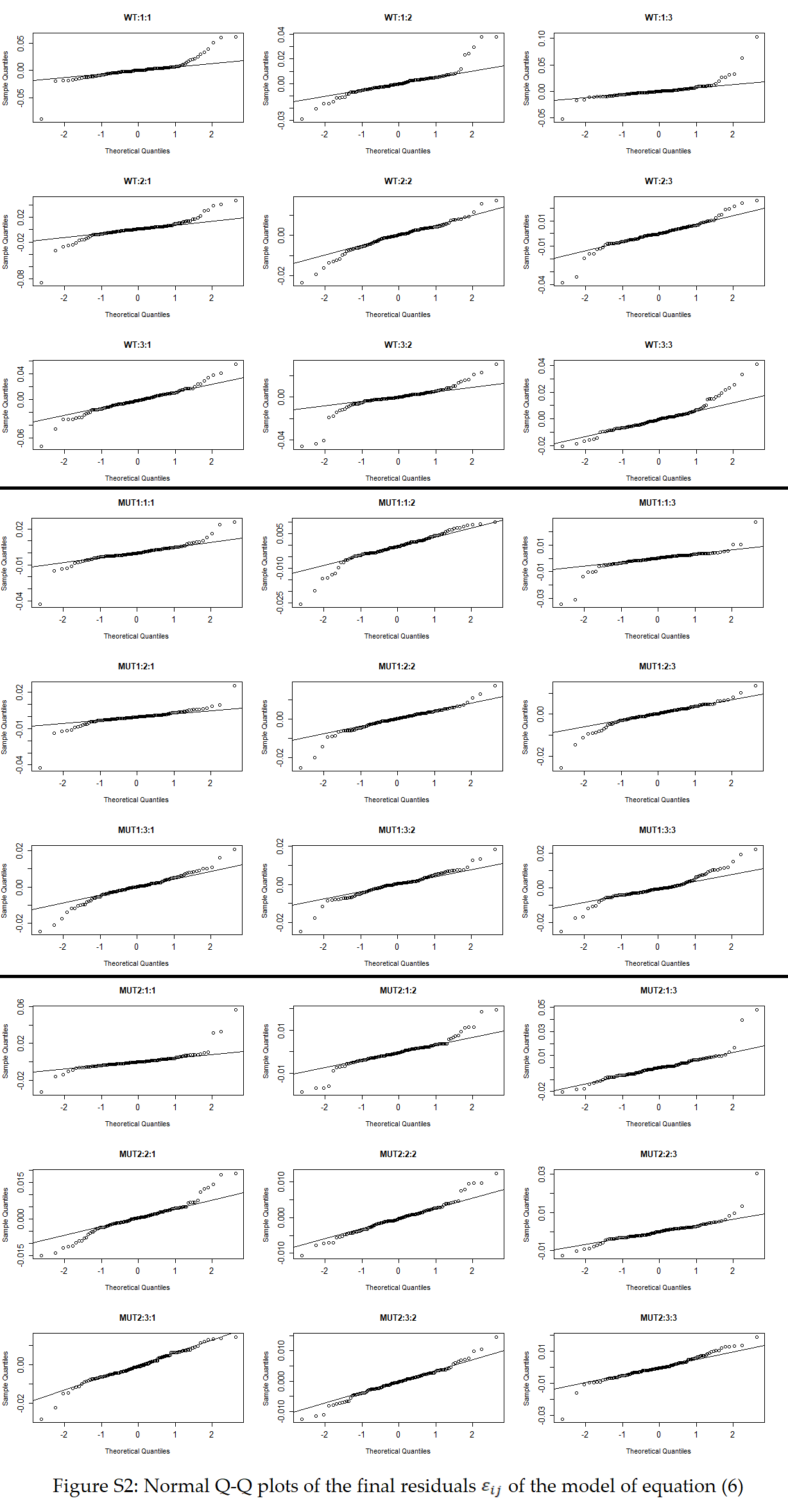

Supplement: Supplementary file 1 [file plants-10-00362-s001.zip › Supplementary material/FigureS2_NEW.png]
